# Prognostic significance of urokinase plasminogen activator and plasminogen activator inhibitor-1 mRNA expression in lymph node- and hormone receptor-positive breast cancer

**DOI:** 10.1186/1471-2407-6-216

**Published:** 2006-08-31

**Authors:** Philippe Leissner, Thibault Verjat, Thomas Bachelot, Malick Paye, Alexander Krause, Alain Puisieux, Bruno Mougin

**Affiliations:** 1 Oncology & Human Genetics Department, bioMérieux SA, Grenoble, F-38024, France; 2 Centre Léon Bérard, Lyon, F-69008, France; 3 INSERM U590, Centre Léon Bérard, Lyon, F-69008, France

## Abstract

**Background:**

One of the most thoroughly studied systems in relation to its prognostic relevance in patients with breast cancer, is the plasminogen activation system that comprises of, among others, the urokinase Plasminogen Activator (uPA) and its main inhibitor, the Plasminogen Activator Inhibitor-1 (PAI-1). In this study, we investigated the prognostic value of *uPA *and *PAI-1 *at the mRNA level in lymph node- and hormone receptor-positive breast cancer.

**Methods:**

The study included a retrospective series of 87 patients with hormone-receptor positive and axillary lymph node-positive breast cancer. All patients received radiotherapy, adjuvant anthracycline-based chemotherapy and five years of tamoxifen treatment. The median patient age was 54 and the median follow-up time was 79 months. Distant relapse occurred in 30 patients and 22 patients died from breast cancer during follow-up. We investigated the prognostic value of *uPA *and *PAI-1 *at the mRNA level as measured by real-time quantitative RT-PCR.

**Results:**

*uPA *and *PAI-1 *gene expression was not found to be correlated with any of the established clinical and pathological factors. Metastasis-free Survival (MFS) and Breast Cancer specific Survival (BCS) were significantly shorter in patients expressing high levels of *PAI-1 *mRNA (p < 0.0001; p < 0.0001; respectively). In Cox multivariate analysis, the level of *PAI-1 *mRNA appeared to be the strongest prognostic factor for MFS (Hazard Ratio (HR) = 10.12; p = 0.0002) and for BCS (HR = 13.17; p = 0.0003). Furthermore, *uPA *gene expression was not significantly associated neither with MFS (p = 0.41) nor with BCS (p = 0.19). In a Cox-multivariate regression analysis, *uPA *expression did not demonstrate significant independent prognostic value.

**Conclusion:**

These findings indicate that high *PAI-1 *mRNA expression represents a strong and independent unfavorable prognostic factor for the development of metastases and for breast cancer specific survival in a population of hormone receptor- and lymph node-positive breast cancer patients.

## Background

The serine protease urokinase-type Plasminogen Activator (uPA) and its inhibitor, the Plasminogen Activator Inhibitor type 1 (PAI-1), are key players in the proteolytic cascade involved in physiological and pathophysiological degradation and remodeling of the extracellular matrix. In the past two decades, study of the uPA and PAI-1 system in human breast cancer has yielded valuable insights. Several international research groups have demonstrated that the protein content of uPA and PAI-1 in the tumor correlates with disease aggressiveness and has a strong prognostic impact on disease-free survival and overall survival in patients with primary breast cancer [1-6]. A pooled analysis of 8,377 breast cancer patients from 18 different data sets further confirmed the strong and independent prognostic value of uPA/PAI-1 [7]. The observation that the prognostic strength of uPA and PAI-1 was substantially influenced by the administration of adjuvant chemotherapy has led the German investigators to hypothesize that these markers might also have important predictive value [8,9]. Several recent analyses indicated that breast cancer patients with high uPA/PAI-1 protein levels derived a significantly greater benefit from adjuvant chemotherapy than patients with low uPA/PAI-1 contents [10,11]. In the multi-center prospective Chemo N0 trial, administration of cyclophosphamide/methotrexate/5-fluorouracil-based chemotherapy led to a substantial reduction in the risk of disease recurrence in patients with high uPA/PAI-1 [8]. In contrast, the correlation between uPA/PAI-1 status and the benefits from adjuvant endocrine therapy appears to be more contradictory [10-13]. Nevertheless, in metastatic breast cancer, retrospective studies showed that elevated uPA or PAI-1 present in the primary tumor are associated with a poor response to later palliative endocrine therapy [14] suggesting that high levels of uPA and/or PAI-1 do reflect an aggressive phenotype that may be overcome by early systemic therapy in the adjuvant setting but not by palliative therapy at a later stage of the disease.

All these studies referred to gene expression at the protein level, as measured by quantitative enzyme immunoassays in cytosols prepared for hormone receptor assays with quality control programs developed by the EORTC Receptor and Biomarker Study Group [15]. However, despite attaining the of highest level of evidence for clinical application, uPA and PAI-1 are not widely used in clinical practice. This is in part due to the growing abandonment of classical biochemical methods for assessing hormone receptors. In the 1990s, an increased number of pathology laboratories started to measure hormone receptors by immunohistochemistry on paraffin sections and biochemical methods were less used, mainly because they are poorly adapted to small tumors, the numbers of which are growing due to widespread breast cancer screening campaigns. The measurement of *uPA *and *PAI-1 *expression at the mRNA level by real-time quantitative Nucleic Acid Sequence-Based Amplification (NASBA) or RT-PCR, which both need very small amounts of RNA and thus allows the analysis of early-stage tumors, may constitute an alternative to the study of the parameters at the protein level.

In spite of adjuvant chemotherapy and endocrine therapy, about 20% of all lymph node-positive, hormone receptor-positive patients suffer from tumor relapse [16]. The risk of disease recurrence should ideally be identified prior to therapeutic decision making, so that the treatment options can be adapted to the patient's prognosis. Unfortunately, beyond HER2 expression, until now, no biological factor allows us to predict in which patients classical chemo and endocrine therapy will be sufficient. Thus, in order to gain insight in this domain, we selected a retrospective series of 87 lymph node-, hormone receptor-positive breast cancer patients who received chemotherapy and endocrine treatment in an adjuvant setting to investigate the prognostic significance of *uPA *and *PAI-1 *mRNA expression.

## Methods

### Patient characteristics

The 87 patients included in the study were diagnosed at the Centre Léon Bérard (Lyon, France) with hormone receptor-positive, axillary lymph node-positive breast cancer between 1992 and 1999. The study was approved by the institutional review board of the hospital. Fresh frozen tissue from the tumor samples was obtained after breast surgery prior to therapy. All patients were free of metastases at the time of diagnosis. The histological subtype was defined according to WHO standards and the histological grade was assessed according to the score of Scarf, Bloom and Richardson (SBR). Steroid receptor status was determined by immunohistochemistry, and tumors were judged to be receptor-positive when >10% of the cells were stained. The median patient age was 54 (ranging from 29–74) and the median follow-up time was 79 months (ranging from 1–153 months). All patients received radiotherapy, adjuvant polychemotherapy and five years of Tamoxifen treatment. The standard chemotherapy regimen consisted of either four cycles of doxorubicin or epirubicin given in combination with cyclophosphamide [4× AC/EC], or six cycles of 5-flourouracil, doxorubicin/epirubicin and cyclophosphamide [6× FAC/FEC]. Distant relapse occurred in 30 patients, and 22 patients died from breast cancer during follow-up. The main patient's characteristics are summarized in Table 1.

### Real-time quantitative RT-PCR analysis

Single strand cDNA was prepared from 200 ng of total RNA using the ThermoScript™ reverse transcriptase system (Invitrogen, California, USA) at 55°C for 60 min followed by RNAse H reaction at 37°C for 20 min in order to remove RNA template. The final RT product was diluted at 1:10 in diethyl pyrocarbonate-treated water and stored at -20°C. Real-time PCR reactions were performed using a LightCycler™ instrument and the Fast-Start™ DNA Master SYBR Green I real-time PCR kit (Roche Diagnostics, Mannheim, Germany). Thermocycling was performed according to the manufacturer's recommendations in a final volume of 20 μl containing 2 mM MgCl_2_, 0.3 μM of each of the required primers and 10 μl of the appropriate cDNA dilution. PCR was carried out with an initial denaturation step of 10 min at 95°C, followed by 40 cycles consisting of 10s at 95°C for denaturation, 10s at 68°C (with a progressive temperature decrease of 0.5°C per cycle, from 68°C to 58°C) for step annealing and 16 s at 72°C for the final extension step. The quantification of uPA and PAI-1 mRNA levels was carried out with regard to the *cyclophilin B *(*PPIB*) housekeeping gene expression, according to the LightCycler™ Relative Quantification Software instructions (Roche Diagnostics, Mannheim, Germany). Four logarithmic dilutions of a linearized plasmid containing the target fragment were prepared in quadruplicate to generate standard curves by plotting the crossing point (Cp) versus the logarithm of the number of copies. Standard dilutions were optimized to cover the relevant concentration range of target and reference RNA in the sample. In every run, water was included as a negative control to check for cross contamination. The specific primer sequences used for real-time PCR were the following: for *uPA *[GenBank: NM_002658]: sense, 5'-CAGGGCATCTCCTGTGCATG-3'(position 1661–1680), antisense, 5'AGCCCTGCCCTGAAGTCGTTA-3' (position 1834–1855); for *PAI-1 *[GenBank: NM_000602]: sense, 5'-GGGCCATGGAACAAGGATGA-3' (position 394–413), antisense, 5'-CTCCTTTCCCAAGCAAGTTG-3' (position 591–610); for *PPIB *[GenBank: NM_000942]: sense, 5'-AGGAGAGAAAGGATTTGGCT-3' (position 231–250), antisense 5'-CAGGCTGTCTTGACTGTCGTGA-3' (position 449–470).

### Statistical analysis

*UPA *and *PAI-1 *mRNA values were compared with clinical, histological and biological features of tumors using appropriate statistical tests. Differences were considered to be statistically significant when p was < 0.05. Metastasis-free Survival (MFS) time (defined as the time from surgery until detection of a distant metastasis) and Breast Cancer specific Survival (BCS) time (defined as the time between the date of surgery and death provoked by cancer) were used as follow-up endpoints. The "distant metastasis" definition did not take into account second cancer or loco-regional relapse and only distant metastasis was considered as an event. Furthermore, MFS does not include deaths without a prior cancer event. For the univariate and multivariate analyses, we investigated the prognostic value of *uPA *and *PAI-1 *mRNA levels by using the median of expression generating "high" and "low" level groups. For each parameter, MFS and BCS were calculated according to the method of Kaplan and Meier [17] and compared using the log-rank test [18]. The results of the multivariate analysis were expressed in terms of hazard ratio derived from the estimated regression coefficients, along with their 95% confidence intervals (CI). Multivariate analyses based on the Cox proportional hazards model [19] were used to identify the most significant factors related to MFS and BCS.

## Results

### RT-PCR analysis of *uPA *and *PAI-1 *gene expression

**Figure 1:**
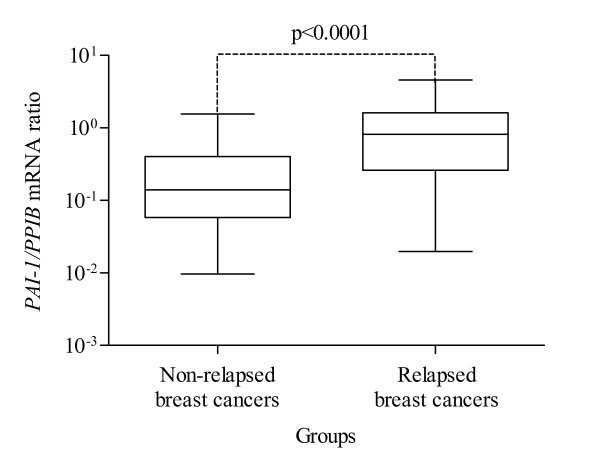
*PAI-1 *mRNA expression in relapsed and non-relapsed breast cancer patients as measured by quantitative RT-PCR. Differential *PAI-1 *gene expression between relapsed (n = 30) and non-relapsed (n = 57) patients with MFS as end-point (Mann-Whitney test; p < 0.0001).

**Figure 2:**
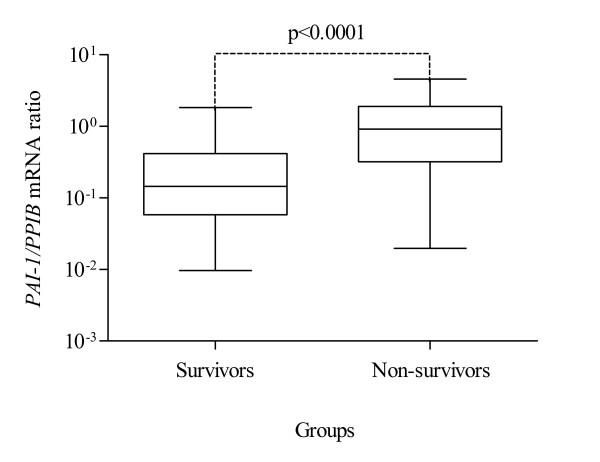
*PAI-1 *mRNA expression in relapsed and non-relapsed breast cancer patients as measured by quantitative RT-PCR. Differential *PAI-1 *gene expression between survivors (n = 65) and non-survivors (n = 22) with BCS as end-point (Mann-Whitney test; p < 0.0001).

Real-time quantitative RT-PCR was carried out to measure *uPA *and *PAI-1 *mRNA expression between relapsed and non-relapsed breast cancers on the 87 samples studied using the *cyclophilin B *(*PPIB*) gene, known to be stably expressed in breast tissues, as an internal control [20]. As shown in Figure 1, significant differential *PAI-1 *gene expression was observed between relapsed and non-relapsed breast cancers (Mann-Whitney p < 0.0001). Similarly, the difference of expression was statistically significant between survivors and patients that died of breast cancer (Figure 2; Mann-Whitney p < 0.0001). In contrast, *uPA *expression levels were not significantly different between breast cancers (data not shown; Mann-Whitney p = 0.09 and p = 0.11 for MFS and BCS, respectively).

### Correlation between *uPA *and *PAI-1 *mRNA values with patient characteristics

The relationship between *uPA/PAI-1 *mRNA levels and patient and tumor characteristics is shown in Table 2. There was no statistically significant correlation between *uPA *mRNA content and age, tumor size, lymph nodes status, age categories, histological types or histological grade. Similarly, the *PAI-1 *expression level was not found to be correlated with any of the established clinical and pathological factors.

### Univariate and multivariate analysis of *uPA *and *PAI-1 *mRNA values for MFS

**Figure 3:**
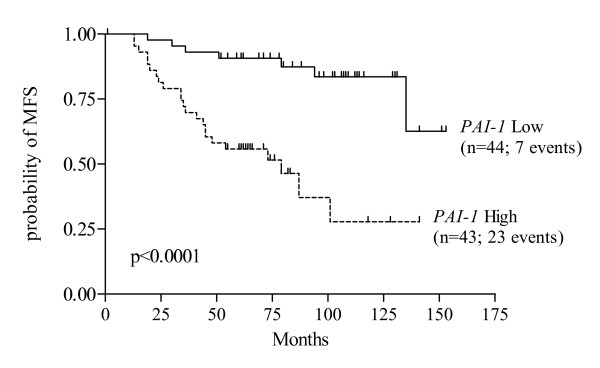
Metastasis-free Survival of patients according to *PAI-1 *gene expression. The high *PAI-1 *expression group (n = 43; 23 events) showed significantly poorer MFS rates than the low *PAI-1 *expression group (n = 44; 7 events; p < 0.0001, log-rank test).

**Figure 4:**
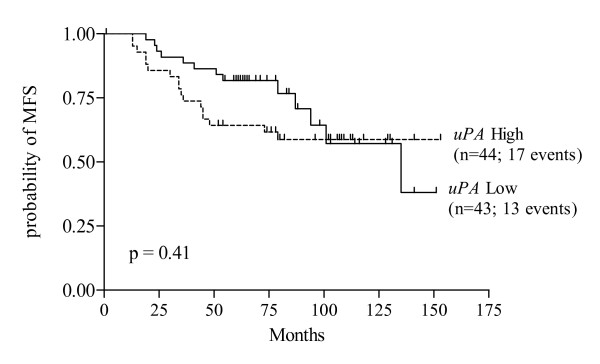
Metastasis-free Survival of patients according to *uPA *gene expression. The high *uPA *expression group (n = 44; 17 events) did not show significantly poorer MFS rates than the low *uPA *expression group (n = 43; 13 events; p = 0.41, log-rank test).

Univariate and multivariate analysis of metastasis-free survival was performed for all parameters. In univariate analysis, we observed that tumor size (p = 0.0002), and the number of lymph nodes involved (p = 0.002) are of prognostic value for MFS (Table 3). Interestingly, *uPA *and *PAI-1 *mRNA expression may also be considered as significant prognostic factors with strong p-values (p = 0.0005 and p < 0.0001 respectively). Kaplan-Meier survival curves representing the probability of metastasis-free survival as a function of *PAI-1 *and *uPA *status are represented in Figures 3 and 4 respectively. We investigated the prognostic value of *uPA *and *PAI-1 *mRNA levels by considering the median of expression as a cut-off generating a "high" and a "low" level group. The medians used corresponded to a *PAI-1/PPIB *and a *uPA/PPIB *expression ratio of 0.25 and 2.12 respectively. As shown in Figure 3, MFS was significantly shorter in patients expressing high level of *PAI-1 *mRNA (p < 0.0001). In contrast, low *uPA *mRNA expression was not significantly associated with longer metastasis-free survival (p = 0.41) (Figure 4).

The independent relationship of *uPA *and *PAI-1 *gene expression with MFS was also studied using Cox-multivariate regression analysis. We could thereby establish whether the prognostic value of each marker as found in the univariate analysis was attributed to its relationship with other clinicopathological factors or whether these factors themselves contribute independently to prognosis. As shown in Table 3, tumor size, age categories and *PAI-1 *mRNA expression are of prognostic value for MFS while lymph node status, histological type, histological grade and *uPA *expression did not add significant independent prognostic information. The tumor size is positively correlated to metastasis-relapse since the risk of relapse is higher for larger tumors than for smaller tumors (p = 0.01). Moreover, the risk of relapse for patients older than 50 years is 3.38 times that found in younger women (p = 0.04). Finally, high levels of *PAI-1 *mRNA appeared to be the strongest prognostic factor with a relative risk of 10.12 (p = 0.0002).

### Univariate and multivariate analysis of *uPA *and *PAI-1 *mRNA values for BCS

**Figure 5:**
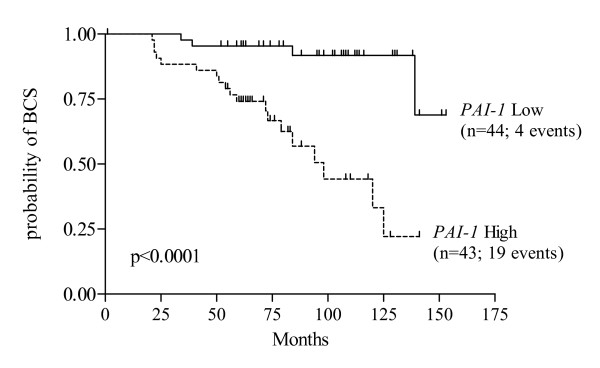
Breast Cancer specific Survival of patients according to *PAI-1 *gene expression. The high *PAI-1 *expression group (n = 43; 19 events) showed significantly poorer BCS rates than the low *PAI-1 *expression group (n = 44; 4 events; p < 0.0001, log-rank test).

**Figure 6:**
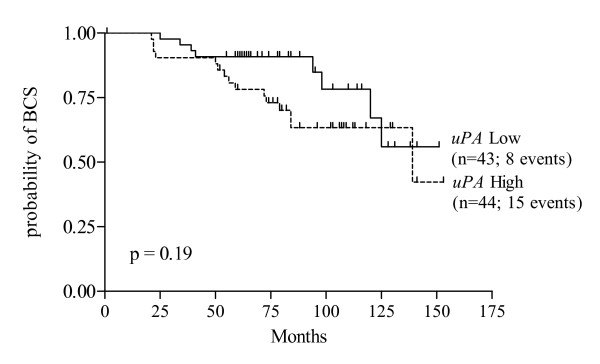
Breast Cancer specific Survival of patients according to *uPA *gene expression. The high *uPA *expression group (n = 44; 15 events) did not show significantly poorer BCS rates than the low *uPA *expression group (n = 43; 8 events; p = 0.19, log-rank test).

Univariate and multivariate analysis were performed using all of the prognostic factors with breast cancer specific survival as the follow-up end point. In univariate analysis, tumor size (p = 0.01), number of lymph nodes involved (p = 0.004), high *uPA *levels (p = 0.02) and high *PAI-1 *levels (p < 0.0001) are of prognostic value for BCS (Table 4). As for *PAI-1*, Kaplan-Meier survival curves representing probability of breast cancer survival as a function of *PAI-1 *and *uPA *status are represented in Figures 5 and 6 respectively. We observed that the probability of BCS was significantly lower in patients expressing a high level of *PAI-1 *mRNA (p < 0.0001; Figure 5) while no significant difference in BCS was observed between patients expressing low and high *uPA *levels (p = 0.19; Figure 6).

In a Cox-multivariate regression analysis, only tumor size (p = 0.05), age categories (p = 0.04), and *PAI-1 *mRNA expression (p = 0.0003) are of prognostic value for BCS while number of lymph nodes, histological type, histological grade and *uPA *expression did not add any significant independent prognostic value (Table 4).

## Discussion

The plasminogen activation system plays a role in cancer progression via extracellular matrix degradation and tumor cell migration [21]. Numerous research groups have demonstrated that the antigen content of uPA and PAI-1 in primary breast cancer tissue correlates with disease aggressiveness and has a strong prognostic impact on primary breast cancer [1-6,21,22]. All these data have been obtained at the protein level using quantitative enzyme-linked immunosorbent assay (ELISA)-based methods applied to cytosol. The EORTC Receptor and Biomarker Study Group initiated quality control programs for this type of assay [23]. However, the use of this method encounters major limitations in routine clinical practice. Although being robust, reproducible and quality-assured, the ELISA requires a substantial amount of frozen tissue, which compromises its use in small tumors (< 1 cm) and requires adequate logistics for the storage of frozen tumor samples. Furthermore, the biochemical methods have been abandoned to the detriment of immunohistochemical methods for hormone receptor assessment in an increasing number of pathology laboratories. Unfortunately, immunohistochemical assays of the uPA/PAI-1 system have provided unsatisfactory results mainly due to an absence of consensus regarding uPA and PAI-1 cellular localization [24]. The measurement of *uPA*- and *PAI-1*-expression at the mRNA level using molecular biology techniques could thus constitute an alternative to the immunochemical assays currently being used. Indeed, real-time quantitative NASBA or RT-PCR needs very low quantities of material (< 100 ng of total RNA) and thus allows for the analysis of smaller tumors. Moreover, the availability of aqueous tissue storage reagents that rapidly permeate tissue to stabilize and protect cellular RNA in unfrozen specimens will facilitate routine laboratory use of those techniques.

Previous studies focused on messengers of the components of the plasminogen activation system in human breast cancer by mainly comparing normal, benign and malignant breast tissues or by examining the cellular localization of *uPA *and *PAI-1 *[25-30]. More recently, Castello *et al*. [31] developed a quantitative real-time RT-PCR assay and showed that *uPA *and *PAI-1 *mRNA expression increased with tumor severity in breast cancer, thereby confirming previous results obtained by Northern blotting [32]. Moreover, it has been demonstrated that high *uPA *and *PAI-1 *mRNA expression was significantly associated with shorter disease-free survival in a population of 130 primary breast cancers independent of the hormone receptor and the lymph node status [33].

To our knowledge, we are the first to evaluate the prognostic impact of *uPA *and *PAI-1 *at the mRNA level in a specific group of lymph node positive- and hormone receptor-positive breast cancers. In the present study, we show that *PAI-1 *mRNA, as measured by quantitative RT-PCR in the primary tumors, has the strongest prognostic value for MFS and BCS. We also observed that the prognostic value of *PAI-1 *is stronger that of *uPA*. Increased *uPA *messenger level was also associated with metastasis-free survival and breast cancer specific survival in univariate analysis, but did not represent a statistically significant independent prognostic factor. The fact that histological grade, one of the most important prognostic factors in hormone receptor-positive breast cancer did not emerge as an independent factor in our analysis was not expected. A major reason might be that we selected a specific population of hormone receptor- and node-positive breast cancer with mainly large tumors. This selection bias might, besides the quite small number of patients, explain these results.

Our results are in accordance with previous studies showing that PAI-1 protein displayed stronger prognostic impact than uPA in lymph node-positive patients and that this marker remained a strong prognostic factor after long-term follow-up both for primary breast cancer and after the first relapse [4,34]. Moreover, it has recently been shown that *PAI-1 *mRNA expression increased with colorectal cancer and the oesophageal squamous carcinoma stage and was associated with poor prognosis suggesting that the gene expression of this marker may serve as a new prognostic factor in these two types of cancer [35,36].

## Conclusion

Taken together, these results demonstrate that a high *PAI-1 *gene expression level represents a strong and independent unfavorable prognostic factor for the development of metastases and for overall survival in a population of lymph node- and receptor-positive breast cancer. In this specific group of patients, the measurement of the *PAI-1 *mRNA level, in addition to the clinicopathological parameters usually considered, may help clinicians to propose a more aggressive chemotherapy (e.g. taxanes instead of anthracyclines) in addition to the endocrine therapy in bad prognosis patients. Moreover, assessing the *PAI-1 *mRNA level may also be useful to stratify candidates for the anti-PAI-1 targeted therapies currently being evaluated [37]. These findings also suggest that for this marker, molecular biology-based techniques such as real-time quantitative NASBA or RT-PCR may be envisaged in the future as an alternative to the ELISA method currently used.

## Competing interests

The author(s) declare that they have no competing interests.

## Authors' contributions

PL conceived of the study and participated in its design and coordination, helped for the statistical analysis and wrote the manuscript. TV carried out the experiments and helped for the statistical analysis. TB conceived of the study and participated in its design and helped to draft the manuscript. MP performed the statistical analysis. AK participate in the preparation of the manuscript and to carry out the statistical analysis. AP conceived of the study and participated in its design and helped to draft the manuscript. BM conceived of the study and participated in its design and coordination and helped to draft the manuscript. All of the authors read and approved the final manuscript.

## Pre-publication history

The pre-publication history for this paper can be accessed here:



## Figures and Tables

**Table 1:** Clinical characteristics of the patients

Characteristics	Total (n = 87)
Age at Diagnosis (years)	
Median (range)	54 (29–74)
Average (SEM)	53 (1.0)
	
Age categories	
< 50 years	30 (35%)
≥ 50 years	57 (65%)
	
Primary surgery	
Mastectomy	48 (55%)
Lumpectomy	39 (45%)
	
Histological type	
Ductal	79 (90%)
Lobular	5 (6%)
Other	3 (4%)
	
Histological grade	
SBR I	12 (14%)
SBR II	47 (54%)
SBR III	28 (32%)
	
Tumor size	
T1	11 (13%)
T2	63 (72%)
T3	13 (15%)
	
Nb. positive nodes	
1–3	55 (63%)
≥ 4	32 (37%)
	
ER status (IHC)	
positive	83 (96%)
negative	4 (4%)
	
PR status (IHC)	
positive	80 (92%)
negative	7 (8%)
	
Cancer-provoked death	
Yes	22 (26%)
No	65 (74%)
	
Metastasis-relapse	
Yes	30 (35%)
No	57 (65%)

Abbreviations: ER, estrogen receptor; PR, progesterone receptor; IHC, immunohistochemistry; SBR, Scarff Bloom and Richardson.

**Table 2:** Association of clinico-pathological factors with *uPA *and *PAI-1 *mRNA expression.

**Variable**	**uPA**	**PAI-1**
	p-value	p-value
	
Age *	0.29	0.3
Tumor size *	0.93	0.95
Lymph nodes *	0.4	0.2
Age categories **	0.2	0.65
< 50 years		
≥ 50 years		
Histological Type **	0.97	1.0
Ductal		
Other		
Histological grade ***	0.76	0.89
SBRI		
SBRII		
SBRIII		

* Spearman rank correlation test

** Wilcoxon rank test

*** One-way ANOVA

Abbreviations: SBR, Scarff Bloom and Richardson.

**Table 3:** Univariate and multivariate analyses for Metastasis-Free Survival

**Variable**	**Univariate**		**Multivariate**	
	p-value	Hazard Ratio (95% CI)	p-value	Hazard Ratio (95% CI)
	
Tumor size	0.0002	1.03 (1.01–1.05)		
T3 vs T1, T2			0.01	9.3 (1.49–58.04)
Lymph nodes	0.002	1.07 (1.02–1.13)		
≥ 3 vs < 3			0.75	1.19 (0.39–3.56)
Age categories	0.08	2.08 (0.89–4.88)		
≥ 50 years vs < 50 years			0.04	3.38 (1.05–10.87)
Histological Type	0.2	3.37 (0.45–24.83)		
Ductal vs others			0.23	4.08 (0.39–42.23)
Histological grade	0.88	1.14 (0.65–2.01)		
SBRII vs I			0.65	1.46 (0.27–7.7)
SBRIII vs I			0.6	1.6 (0.27–9.5)
uPA High vs Low	0.0005	1.14 (1.05–1.24)	0.62	1.31 (0.44–3.93)
PAI-1 High vs Low	<0.0001	5.56 (2.32–13.28)	0,0002	10.12 (3.03–33.76)

Abbreviations: SBR, Scarff Bloom and Richardson.

**Table 4:** Univariate and multivariate analyses for Breast Cancer specific Survival

**Variable**	**Univariate**		**Multivariate**	
	
	p-value	Hazard Ratio (95% CI)	p-value	Hazard Ratio (95% CI)
	
Tumor size	0.01	1.02 (1–1.05)		
T3 vs T1, T2			0.05	6.5 (0.99–42.58)
Lymph nodes	0.004	1.08 (1.02–1.15)		
≥ 3 vs < 3			0.77	1.19 (0.35–4.02)
Age categories	0.29	1.64 (0.64–4.18)		
≥ 50 years vs < 50 years			0.04	3.63 (1.04–12.6)
Histological Type	0.14	1.05 (0.72–1.25)		
Ductal vs others			0.96	1.65 (0.3–4.56)
Histological grade	0.85	1.04 (0.53–2.03)		
SBRII vs I			0.68	0.69 (0.12–3.97)
SBRIII vs I			0.57	0.57 (0.08–3.83)
uPA High vs Low	0.02	1.11 (1.01–1.23)	0.13	2.57 (0.74–8.9)
PAI-1 High vs Low	<0.0001	7.61 (2.55–22.71)	0.0003	13.17 (3.26–53.17)

Abbreviations: SBR, Scarff Bloom and Richardson.
